# Multi-omics analysis of gut microbiota and metabolites reveals the effects of rearing systems on the duodenum and cecum gut barriers of lueyang black-bone chickens

**DOI:** 10.3389/fmicb.2025.1642169

**Published:** 2025-09-03

**Authors:** Linqing Shao, Mingming Zhao, Shuang Zeng, Ling Wang, Shanshan Wang, Wenxian Zeng, Hongzhao Lu

**Affiliations:** ^1^School of Biological Science and Engineering, Shaanxi University of Technology, Hanzhong, China; ^2^Engineering Research Center of Quality Improvement and Safety Control of Qinba Special Meat Products, Universities of Shaanxi Province, Hanzhong, China; ^3^QinLing-Bashan Mountains Bioresources Comprehensive Development C. I. C., Shaanxi University of Technology, Hanzhong, China

**Keywords:** Lueyang black-bone chicken, gut barrier, rearing systems, microbial diversity, metabolomics

## Abstract

**Introduction:**

Gut health is a critical determinant of poultry growth, immunity, and meat quality, with the intestinal barrier being fundamental to its maintenance. This study aimed to investigate the impacts of caged and cage-free rearing systems on the gut barrier of Lueyang black-bone chickens, specifically focusing on how these systems alter gut microbiota composition and metabolic profiles.

**Methods:**

Lueyang black-bone chickens were raised under either caged or cage-free conditions. Gut barrier integrity was assessed through histological examination of the duodenum and cecum. Microbial community structure was analyzed via 16S rRNA sequencing, and metabolic changes were profiled using LC–MS-based non-targeted metabolomics.

**Results:**

Histological analysis revealed significantly greater intestinal wall thickness and higher goblet cell counts in the cage-free group (*p* < 0.0001). Microbiome sequencing showed that the caged group was enriched with fiber-degrading bacteria such as *Bacteroides* and *Rikenellaceae_RC9_gut_group*, while the cage-free group had a higher abundance of potential pathogens like *Acinetobacter* and *Campylobacter*. Metabolomics results indicated upregulation of bile acids and unsaturated fatty acids in the caged group, whereas phosphatidylcholine (PE) and lysophosphatidylcholine (LPC) were significantly elevated in the cage-free group. Further integrated analysis revealed strong positive correlations between Rikenellaceae_RC9_gut_group and bile acids, and between *Odoribacter*/*Clostridia_UCG-014* and unsaturated fatty acids including traumatic acid.

**Discussion:**

The findings suggest that caged rearing promoted a more beneficial microbial community structure, characterized by fiber-degrading bacteria that subsequently elevated anti-inflammatory and barrier-strengthening metabolites such as bile acids and unsaturated fatty acids. In contrast, the cage-free environment may predispose chickens to potential gut inflammation and barrier dysfunction, partly linked to higher levels of harmful bacteria and membrane phospholipid metabolites. These results highlight the role of rearing systems in modulating gut health through microbiota-metabolite interactions.

## 1 Introduction

Lueyang black-bone Chicken is a local Chinese breed characterized by its large size, strong disease resistance, tender meat, and exceptional antioxidant activity. For instance, antioxidant peptides, such as Glu-Pro-Asp-Arg-Tyr and myostatin, present in Lueyang black-bone Chicken meat, may alleviate atherosclerosis, diabetes mellitus, and neurodegenerative diseases by scavenging free radicals, chelating metal ions, and through other mechanisms (Elias et al., 2008; Liu et al., 2013). These characteristics are closely associated with the rearing systems of Lueyang black-bone Chicken. Different rearing systems affect the growth performance and health of Lueyang black-bone Chicken by altering the growing environment and nutritional supply (Bai et al., 2022). Although traditional cage-free rearing meets animal welfare needs, reliance on natural foraging can result in imbalanced calcium and phosphorus metabolism. Additionally, the infection rate of parasites, such as coccidia, is significantly higher in cage-free rearing flocks compared to caged ones (Chen et al., 2018), potentially causing gut damage and growth retardation. Although caged rearing improves feed conversion rates, long-term bedding accumulation may promote the growth of pathogenic microorganisms, such as *Escherichia coli* and Salmonella spp., leading to gut inflammation (Abo Ghanima et al., 2020; Burrows et al., 2025). Therefore, exploring rearing systems that maintain production performance while safeguarding gut health has become a pressing issue in the industry.

Studies have demonstrated that rearing systems can regulate the structure and diversity of the microbiota in the cecum, thereby indirectly influencing host health. In pig farming, the abundance of beneficial bacteria, such as *Bifidobacteria*, in the cecum of cage-free reared pigs is significantly higher than that in intensively reared pigs. In contrast, the proportion of potentially pathogenic bacteria, such as *Clostridium perfringens*, is reduced by 50% (Qi et al., 2022). Marcolla C. S. et al. reported that cage-free reared broilers exhibited higher microbial community diversity in their cecums, with significantly greater abundance of *Bacteroides* and *Clostridium* compared to caged reared broilers. This may be attributed to the fact that, in intensive farming systems, confined spaces and standardized feed shorten chyme retention time, promoting the overgrowth of *Clostridium perfringens* and impairing gut health (Marcolla et al., 2023). However, existing research has largely overlooked microbial diversity in the duodenum. The duodenum, as the initial segment of the small intestine, is the primary region for nutrient digestion (Scaldaferri et al., 2012). The duodenum, located adjacent to digestive glands with a pH < 7.0, has an acidic environment that inhibits the growth of harmful bacteria and promotes the colonization of beneficial bacteria (Smet et al., 2015). For instance, in the duodenum, lactic acid bacteria convert carbohydrates into lactic acid via lactate dehydrogenase secretion, lowering the local pH and inhibiting *Salmonella* colonization (Liu et al., 2019; Schroeder, 2019). The density of goblet cells in the duodenum is 2.3 times greater than in the cecum. The mucus secreted by goblet cells, MUC2, forms a dense layer that physically isolates microorganisms from epithelial cells (Schroeder, 2019; Paone and Cani, 2020). The core functional microbiota, *Enterococcus*, induces mucus glycosylation modification to enhance the pathogen barrier function. Additionally, bile salt hydrolase (BSH) encoded by *Enterococcus* separates bound bile acids, promotes lipid digestion, and directly affects nutrient utilization (Alenezi et al., 2024). Therefore, the role of the duodenum and its microbiota in maintaining gut health must not be overlooked.

The intestinal barrier consists of mechanical, chemical, microbial, and immune components (Camilleri et al., 2012; Paone and Cani, 2020). Damage to the intestinal barrier may allow pathogens like *Escherichia coli* and Salmonella to penetrate the intestinal mucosa and induce inflammation (Williams, 2005). Studies have demonstrated that the synergistic action of the microbiota across different gut segments plays a critical role in maintaining the function of the gut barrier. The dominant *Lactobacilli* in the duodenum produce lactic acid through homofermentative fermentation, lowering the pH to 4.5–5.0, thereby inhibiting the type III secretion system (T3SS) of pathogens, such as *Salmonella*, and reducing their ability to secrete the effector protein SopE, thus preventing damage to the physical barrier (Boddy et al., 2021; Hussain et al., 2021). Furthermore, antimicrobial peptides secreted by *Lactobacilli* can penetrate cell membranes, interfere with DNA replication and cell wall protein synthesis, thereby directly blocking pathogen proliferation (Smith and Hillman, 2008). The cecal microbiota produces metabolites, such as short-chain fatty acids (SCFAs), through the fermentation of undigested dietary fiber. These metabolites bind to GPR43 receptors on the surface of intestinal epithelial cells, induce membrane hyperpolarization by stimulating potassium (K+) flow, activate NLRP3 inflammasomes, and promote the secretion of IL-1/IL-18, thus enhancing intestinal barrier integrity and reducing inflammation (Ghosh et al., 2021). Additionally, butyrate, a metabolite of the cecal microbiota, increases the expression of the Muc2 and SPDEF genes in goblet cells via M2-type macrophages, activates the M2/WNT/ERK signaling pathway, and promotes intestinal mucus barrier repair (Gasaly et al., 2021). Therefore, systematic studies of the structural and metabolic changes in the cecal and duodenal microbiota are crucial for understanding how rearing systems impact the gut barrier health of Luoyang black-boned chickens.

While studies have highlighted the importance of rearing systems in influencing the diversity of gut flora in poultry, systematic studies across gut segments in Lueyang black-bone Chicken, a local specialty breed, are still lacking. Most studies have focused solely on structural changes in gut flora, overlooking changes in gut barrier function and its interactions with flora metabolism. In this study, 16S rRNA technology and non-targeted metabolomics were combined to systematically analyze the gut morphology, gut flora structure, and metabolic profiles of the cecum and duodenum in Lueyang black-bone Chicken under cage-free and caged rearing modes. Spearman correlation analysis was used to identify core bacterial genera and metabolic markers that could improve barrier function. The results of this study provide a theoretical basis for optimizing healthy breeding modes for Lueyang black-bone Chicken and offer new insights for improving gut health and meat quality in local chicken breeds through targeted bacterial flora interventions. This has significant practical value for promoting the development of green animal husbandry.

## 2 Materials and methods

### 2.1 Ethics approval statement

All animal experimental procedures in this study were approved and implemented following the guidelines of the Animal Care and Utilization Committee of Shaanxi University of Technology (2025031803).

### 2.2 Animal resources and rearing systems

This experiment was conducted at the Shaanxi Longjia Agricultural Technology Co., Ltd. (Lueyang County, Shaanxi Province) breeding farm. Before the experiment, 300 one-day-old female Lueyang black-bone chicks were raised in cages within the same brood house until they reached 30 days old. Subsequently, 300 hens were isolated and randomly allocated to two groups: caged rearing (CR) or cage-free rearing (CF). Each group contained 3 replicates, with 50 chickens in each replicate. For the CR groups, two chickens were kept in each conventional cage, and every chicken had an average floor area of 0.15 m^2^. The cage system was environmentally controlled, in which the average temperature was 20–25°C, the relative humidity was 65–70%, and the indoor photoperiod was 16:8 light: dark. For the CF groups, chickens were fed in an indoor floor house (bird/0.15 m^2^) with an adjacent outdoor cage-free area (bird/1 m^2^). The indoor environmental conditions were controlled similarly to those of the CR group, and the outdoor average temperature was 20–25°C in the local mountain areas during the formal experiment. All chickens had unrestricted access to feed and water, with identical feed formulations and nutrient levels in both groups (Zhang et al., 2022).

### 2.3 Sample collection

At 3 months of age, 6 chickens from each group were randomly selected and euthanized using carbon dioxide anesthesia and cervical dislocation. Histological specimens were taken from the duodenum and cecum, and tissue samples were fixed with 10% phosphate-buffered formalin for HE staining. To analyze gut metabolites and microbiota, 3–5 g of duodenal and cecal contents were scraped from each group, placed in sterilized 1.5 mL centrifuge tubes, rapidly frozen in liquid nitrogen, and stored at −80°C until sequencing.

### 2.4 Gut morphology measurement

Assess the histomorphology of duodenal and cecal tissues using hematoxylin and eosin (H&E) staining. Three animals were randomly selected from each group of healthy animals with similar body weights, euthanized, and their guts were quickly dissected and collected. The guts were gently rinsed with sterilized PBS buffer solution, cut into 2 cm long pieces, placed in a centrifuge tube containing 8 mL of 4% paraformaldehyde fixative, and fixed at room temperature for 24 h. After dehydration, clearing, wax immersion, and embedding, the duodenum and cecum were sectioned using a microtome and stained with hematoxylin and eosin. The intestinal tissue structure of each group was observed using an Olympus BX53 upright microscope, and the intestinal wall thickness, goblet cell number, villus height (VH), and crypt depth (CD) were measured. Three sections were selected for measurement, and the average values were calculated.

### 2.5 DNA extraction

According to the manufacturer’s instructions, total microbial genomic DNA was extracted from Duodenum and cecum samples using the FastPure Feces DNA Isolation Kit (Shanghai Major Yuhua). The quality and concentration of DNA were determined by 1.0% agarose gel electrophoresis and a NanoDrop2000 spectrophotometer (Thermo Scientific, United States) and kept at −80°C before further use.

### 2.6 High-throughput 16S rRNA gene sequencing

The hypervariable region V3-V4 of the bacterial 16S rRNA gene was amplified with primer pairs 338F (5’-ACTCCTACGGGAGGCAGCAG-3′) and 806R (5’-GGACTACHVGGGTWTCTAAT-3′)(Liu et al., 2016) by T100 Thermal Cycler PCR thermocycler (BIO-RAD, United States). The reaction system included 4 μL of 5 × FastPfu Buffer, 2 μL of 2.5 mM dNTPs, 0.8 μL of each primer (5 μM), 0.4 μL of FastPfu Polymerase, and 10 μL of DNA template. The processes were as follows: the denaturation lasted for 3 min at 95°C followed by 27 cycles of 95°C for 30 s, 55°C for 30 s, and 72°C for 45 s, with a final extension of 10 min at 72°C. Meanwhile, the PCR product was extracted from 2% agarose gel, purified using the PCR Clean-Up Kit (YuHua, Shanghai, China) according to the manufacturer’s instructions, and quantified using Qubit 4.0 (Thermo Fisher Scientific, United States).

### 2.7 Sequencing data analysis

Bioinformatic gut microbiota analysis was carried out using the Majorbio Cloud platform. 16S rRNA gene data from 24 samples (the cecum and duodenum of six caged and six cage-free reared Lueyang black-bone chickens) were analyzed using the classification-sklearn classifier to classify and annotate representative Amplicon Sequence Variants (ASVs) via the SILVA 16S rRNA database. To facilitate downstream diversity and composition analyses, sequences from each sample were normalized based on the minimum number of sequences per sample, yielding 726,480 valid sequences. Then, the high-quality sequences were de-noised using the DADA2 plugin in the Qiime2 (version 2020.2) pipeline with recommended parameters, which obtains single-nucleotide resolution based on error profiles within samples. DADA2-denosed sequences are typically called amplicon sequence variants (ASVs) (Callahan et al., 2016). To minimize the effects of sequencing depth on alpha and beta diversity measures, the number of sequences from each sample was rarefied to 20,000, which still yielded an average Good’s coverage of 97.90%. Taxonomic assignment of ASVs was performed using the Naive Bayes consensus taxonomy classifier implemented in Qiime2 and the SILVA 16S rRNA database (v138). Based on the ASVs’ information, rarefaction curves and alpha diversity indices, including observed ASVs, Chao1 richness, Shannon index, and Good’s coverage, were calculated with Mothur v1.30.1 (Schloss et al., 2009). The similarity among the microbial communities in different samples was determined by principal coordinate analysis (PCoA) based on Bray–Curtis dissimilarity using the Vegan v2.5–3 package. The PERMANOVA test was used to assess the percentage of variation explained by the treatment and its statistical significance using the Vegan v2.5–3 package. The linear discriminant analysis (LDA) effect size (LEfSe)^1^ was performed to identify the significantly abundant taxa (phylum to genera) of bacteria among the different groups (LDA score > 3, *p* < 0.05). Correlation analysis employs the MajorBio cloud platform to analyze relationships between differential metabolites and the differential microbiota, thereby assessing the strength of the association between these two variables. A correlation between two nodes was considered statistically robust if the Spearman’s correlation coefficient was over 0.6 or less than −0.6, and the *p*-value was less than 0.01 (Barberán et al., 2012). To control the risk of false positives caused by multiple hypothesis testing, use the Benjamini-Hochberg false discovery rate (FDR) procedure to adjust *p*-values for multiple comparisons. The significance threshold was set at FDR < 0.05.

### 2.8 Metabolite extraction and quality control sample

Accurately weigh 50 ± 5 mg of gut sample into a 2 mL centrifuge tube, add 400 μL of extraction solution [methanol: water = 4:1 (v/v)] containing 0.02 mg/mL of internal standard (L-2-chlorophenylalanine) to extract the metabolites. The sample solution was ground in a cryogenic tissue grinder for 6 min (−10°C, 50 Hz), followed by low-temperature ultrasonic extraction for 30 min (5°C, 40 kHz). The sample was then left to stand at −20°C for 30 min, centrifuged for 15 min (4°C, 13,000 g), and the supernatant was transferred. Additionally, 20 μL of the supernatant was transferred, mixed, and used for LC–MS/MS analysis. As a part of the system conditioning and quality control process, a pooled quality control sample (QC) was prepared by mixing equal volumes of all samples. The QC samples were disposed of and tested in the same manner as the analytical samples. It helped to represent the whole sample set, which would be injected at regular intervals (every 5–15 samples) to monitor the stability of the analysis.

### 2.9 LC–MS/MS analysis and data processing

The LC–MS/MS analysis of the sample was conducted on a Thermo UHPLC-Q Exactive HF-X system equipped with an ACQUITY HSS T3 column (100 mm × 2.1 mm i.d., 1.8 μm; Waters, United States) at Majorbio Bio-Pharm Technology Co. Ltd. (Shanghai, China). The mobile phases consisted of 0.1% formic acid in water: acetonitrile (95:5, v/v) (solvent A) and 0.1% formic acid in acetonitrile:isopropanol: water (47.5:47.5, v/v) (solvent B). The flow rate was 0.40 mL/min, and the column temperature was 40°C. The mass spectrometric data were collected using a Thermo UHPLC-Q Exactive HF-X Mass Spectrometer with an electrospray ionization (ESI) source operating in positive and negative modes. The optimal conditions were set as followes: source temperature at 425°C; sheath gas flow rate at 50 arb; Aux gas flow rate at 13 arb; ion-spray voltage floating (ISVF) at -3500 V in negative mode and 3,500 V in positive mode, respectively; Normalized collision energy, 20–40-60 V rolling for MS/MS. Full MS resolution was 60,000, and MS/MS resolution was 7,500. Data acquisition was performed in Data Dependent Acquisition (DDA) mode. The detection was carried out over a mass range of 70–1,050 m/z. The solvent gradients of positive ion mode were changed according to the following conditions: from 0 to 3 min, solvent B 0–20%; from 3 to 4.5 min, solvent B 20–35%; from 4.5 to 5 min, solvent B 35–100%; from 5 to 6.3 min, solvent B 100–100%; from 6.3 to 6.4 min, solvent B 100–0%; from 6.4 to 8 min, solvent B 0–0% for equilibrating the systems. As for the negative ion mode, the operational conditions were from 0 to 1.5 min, solvent B 0–5%; from 1.5 to 2 min, solvent B 5–10%; from 2 to 4.5 min, solvent B 10–30%; from 4.5 to 5 min, solvent B 30–100%; from 5 to 6.3 min, solvent B 100–100%; from 6.3 to 6.4 min, solvent B 100–0%; from 6.4 to 8 min, solvent B 0–0% for equilibrating the systems. The pretreatment of LC/MS raw data was performed by Progenesis QI (Waters Corporation, Milford, United States) software, and a three-dimensional data matrix in CSV format was exported. This three-dimensional matrix included: sample information, metabolite name, and mass spectral response intensity. Internal standard peaks and any known false positive peaks (including noise, column bleed, and derivatized reagent peaks) were removed from the data matrix, deduplicated, and peak-pooled. At the same time, the metabolites were identified by searching the database, and the central databases were the HMDB V5.0,^2^ METLIN 2019 MS/MS Library,^3^ and Majorbio Database. The preprocessed data were analyzed using the ropls package (Version 1.6.2) in R for principal component analysis (PCoA) and orthogonal partial least squares discriminant analysis (OPLS-DA) on the preprocessed data matrix, and the stability of the model was evaluated using 7-fold cross-validation. Significantly different metabolites were selected based on the variable importance in projection (VIP) values obtained from the OPLS-DA model and the *p*-values from Student’s t-test. Metabolites with VIP > 1 and *p* < 0.05 were considered significantly different metabolites. Differential metabolites were annotated for metabolic pathways using the KEGG database,^4^ identifying the pathways in which differential metabolites were involved. Pathway enrichment analysis was performed using the Python package scipy.stats.

### 2.10 Statistical analysis

All experimental data analysis was performed using SPSS software (version 26.0), and graphs were generated using GraphPad Prism 8.0 software. The Shapiro–Wilk test was used to evaluate the normality of the data. On this basis, an independent samples t-test was conducted to compare the differences between the two groups, and *p* < 0.05 indicated a significant difference (*), *p* < 0.01 indicated a highly significant difference (**), *p* < 0.001 indicated an extremely significant difference (***), and < 0.0001 indicated an extremely significant difference (****).

## 3 Results

### 3.1 Effects of different rearing systems on the gut morphology of the cecum and duodenum in Lueyang black-bone chickens

**Figure 1:**
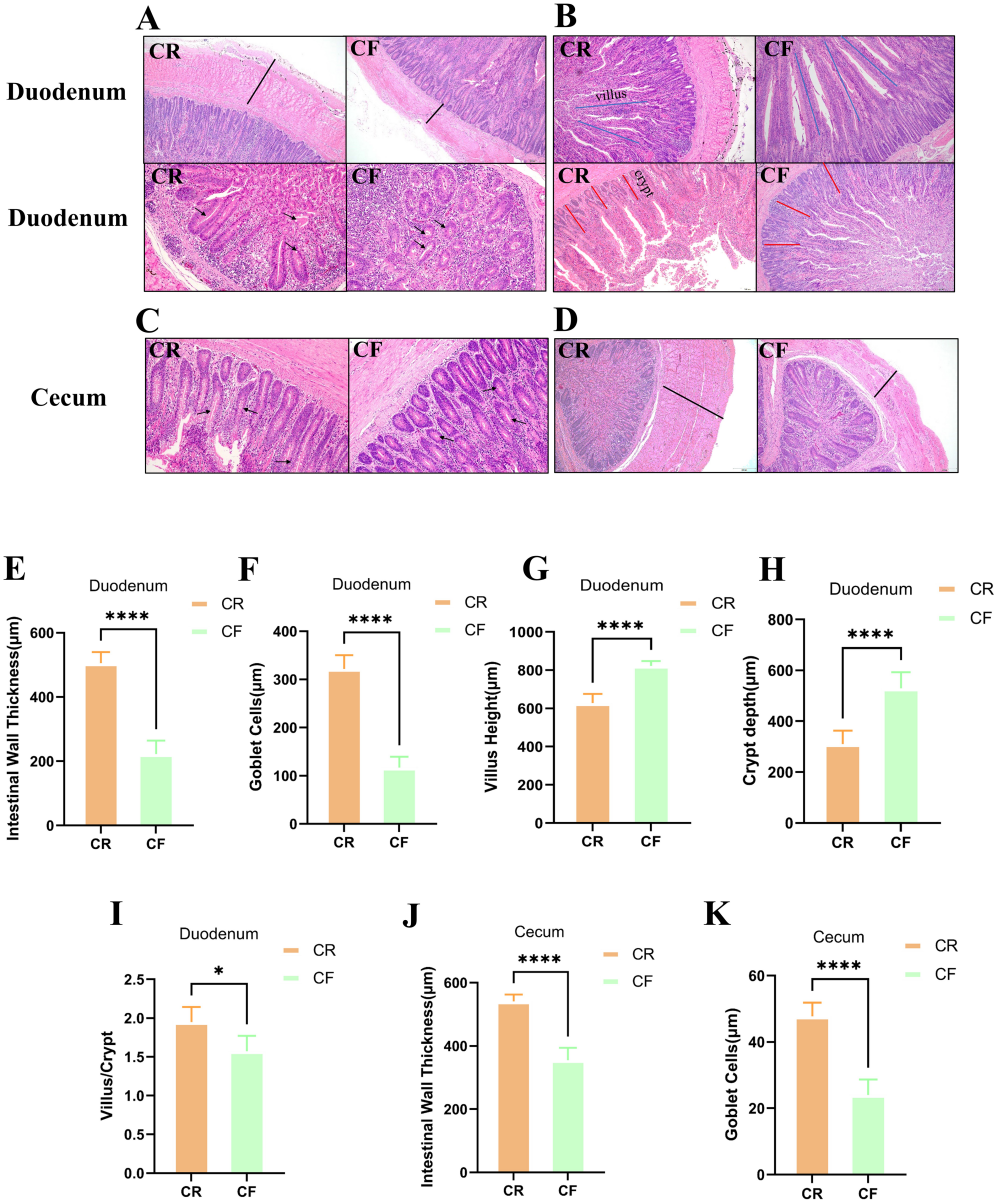
HE-stained sections of various intestinal segments under different rearing systems. **(A–D)** HE staining of duodenal and cecal tissue sections from Lueyang black-bone chickens. Samples were taken at week 12. The black arrows in **(A,C)** indicate goblet cells; the black lines in **(A,D)** denote intestinal wall thickness; the blue lines in **(B)** represent villus height; and the red lines in **(B)** represent crypt depth. Statistical analysis was performed using the t-test; *p* > 0.05, *p < 0.05, **p < 0.01, ***p < 0.001, ****p < 0.0001; **(E–H)** Duodenal intestinal wall thickness and goblet cell count; **(I)** Ratio of villus length to crypt depth in the duodenum; **(J–K)** Intestinal wall thickness and goblet cell count in the cecum.

To explore the effect of rearing systems on the gut morphology of Lueyang black-bone chickens, histological sections were subjected to HE staining. The morphological characteristics of the duodenum and cecum of the chickens are illustrated in Figures 1A–D. In the duodenum, the intestinal wall thickness and the number of goblet cells were significantly lower in the cage-free rearing group compared to the caged rearing group (Figures 1E,F; *p* < 0.0001). In the cage-free rearing group, the goblet cells in the intestinal mucosa were significantly reduced and scattered. In contrast, the goblet cells in the intestinal mucosa of the caged rearing group were round or oval, numerous, and visible, with no apparent defects or deficiencies. They were densely distributed throughout the villi. Furthermore, by measuring the height of the intestinal villi (VH) and crypt depth (CD) and calculating the VH/CD ratio, it was found that compared with the caged rearing group, the villus height and crypt depth of the cage-free rearing group showed a significant increase (Figures 1G,H; *p* < 0.0001), but the ratio of villus height to crypt depth was significantly lower than that of the caged rearing group (Figure 1I; *p* < 0.05). These results indicate that the cage-free rearing group had an enhanced gut absorption area and may have had stronger digestion and absorption of nutrients. However, their gut barrier was weaker than that of the caged rearing group. In the cecum, the morphological structure of the intestinal mucosa in the caged rearing group remained unchanged, with the intestinal wall thickness significantly higher than that of the cage-free rearing group (Figure 1J; *p* < 0.0001). In addition, compared to the caged rearing group, the cage-free rearing group exhibited marked cell infiltration and edema, significantly reducing the number of goblet cells (Figure 1K; *p* < 0.0001).

### 3.2 Different rearing systems affect the abundance and diversity of gut microbiota in Lueyang black-bone chickens

**Figure 2:**
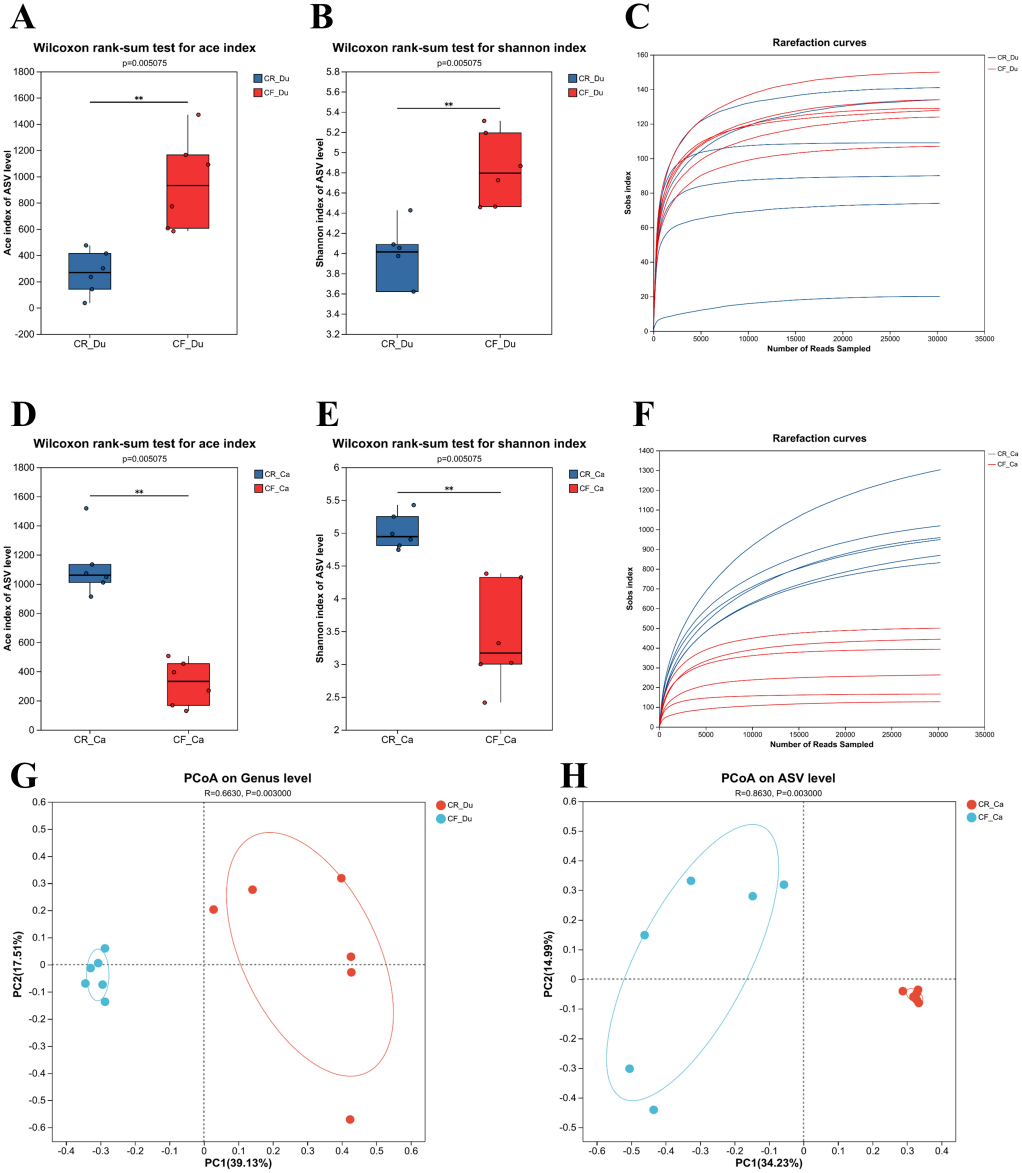
Intergroup differences in cecum and duodenum indices, dilution curves, and PcoA analysis under different rearing systems. **(A–C)** Comparison of ACE and Shannon indices between the two groups of duodenum samples, along with dilution curves; **(D–F)** Comparison of ACE and Shannon indices between the two groups of cecum samples, along with dilution curves; **(G,H)** PCoA analysis based on Bray–Curtis distance.

To analyze the abundance and diversity of the gut microbiota in Lueyang black-bone chickens, *α* diversity index and *β* diversity analyses were performed. In the duodenum, the ACE and Shannon indices of the gut microbiota in caged Lueyang black-bone chickens decreased significantly, indicating lower abundance and diversity compared to cage-free reared chickens (Figures 2A,B; *p* < 0.05). In the cecum, the ACE and Shannon indices of the gut microbiota in caged Lueyang black-bone chickens showed a significant increase. This suggests that the abundance and diversity of the gut microbiota in caged Lueyang black-bone chickens were higher than in cage-free reared chickens (*p* < 0.05; Figures 2D,E). Dilution curve results showed that as the number of extracted sequences increased, the curve flattened, indicating that the sequencing data volume was adequate (Figures 2C,F). Bray-Curtis PCoA analysis showed complete separation of the cage-free and caged rearing groups along the PC1 axis in both the duodenum and cecum (Figures 2G,H), indicating significant changes in the gut microbiota of Lueyang black-bone chickens under different rearing systems (*p* < 0.01).

### 3.3 Impact of different rearing systems on the community composition of Lueyang black-bone chickens

**Figure 3:**
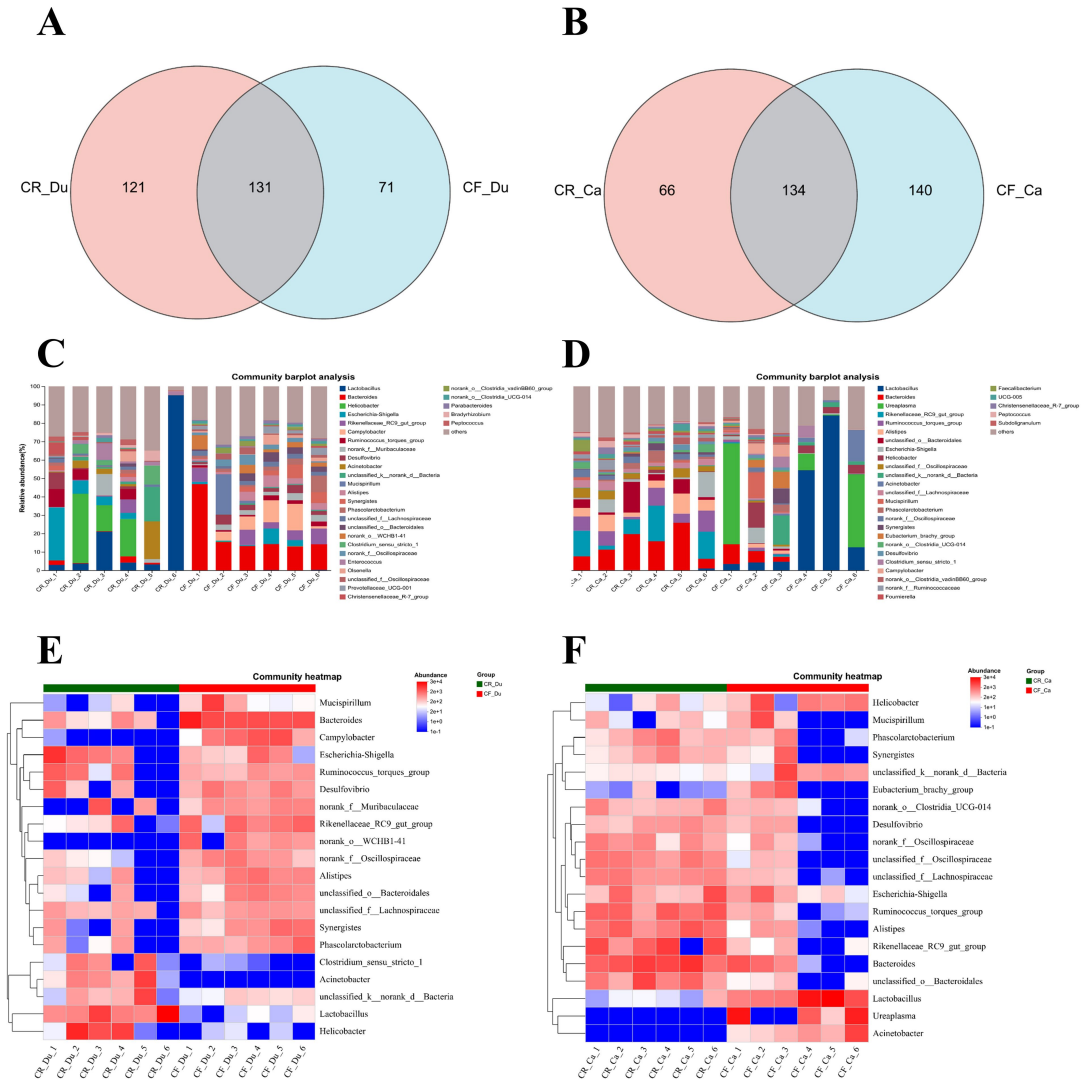
Effects of different rearing systems on the community composition of Lueyang black-bone chickens. **(A,B)** Venn diagram analysis of the duodenal and cecal microbiota in Lueyang black-bone chickens; **(C,D)** Bar chart of the community composition of the duodenum and cecum microbiota in Lueyang black-bone chickens at the genus level, with data presented as means (*n* = 8); **(E,F)** Heatmap of the duodenum and cecum microbiota in Lueyang black-bone chickens.

The community composition of each sample was studied by clustering all samples using ASVs (Amplicon Sequence Variants) and plotting the results as a Venn diagram. In the duodenum, 71 species were unique to cage-free Lueyang black-bone chickens, while 121 species were unique to caged-reared chickens, with 131 species common to both groups (Figure 3A). In the cecum. There were 140 bacterial genera in cage-free reared Lueyang black-bone chickens absent in caged chickens. 66 bacterial genera in caged Lueyang black-bone chickens were absent in cage-free reared chickens. In addition, there were 134 species common to both rearing systems (Figure 3B). These results indicate that the gut microbiota of Lueyang black-bone chickens changes when they transition from a natural, cage-free environment to an artificial, restricted caged rearing system.

The community bar chart and heatmap display the bacterial community composition at the genus level in both caged and cage-free reared Lueyang black-bone chickens. The comprehensive community bar chart and heatmap show that in the duodenum, the dominant genera in the caged rearing group were *Lactobacillus*, *Acinetobacter*, *Helicobacter*, and *Escherichia-Shigella*. The dominant bacterial genera in the cage-free rearing group were *Bacteroides*, *Rikenellaceae_RC9_gut_group*, *Campylobacter*, and *Desulfovibrio* (Figures 3C,E). In the cecum, the dominant bacterial genera in the caged rearing group were *Bacteroides*, *Rikenellaceae_RC9_gut_group*, *Ruminococcus_torques_group*, *Alistipes*, and *Escherichia-Shigella*. The dominant bacterial genera in the cage-free rearing group were *Lactobacillus*, *Ureaplasma*, *Helicobacter*, and *Acinetobacte* (Figures 3D,F).

### 3.4 Species differences in the gut microbiota of Lueyang black-bone chickens

**Figure 4:**
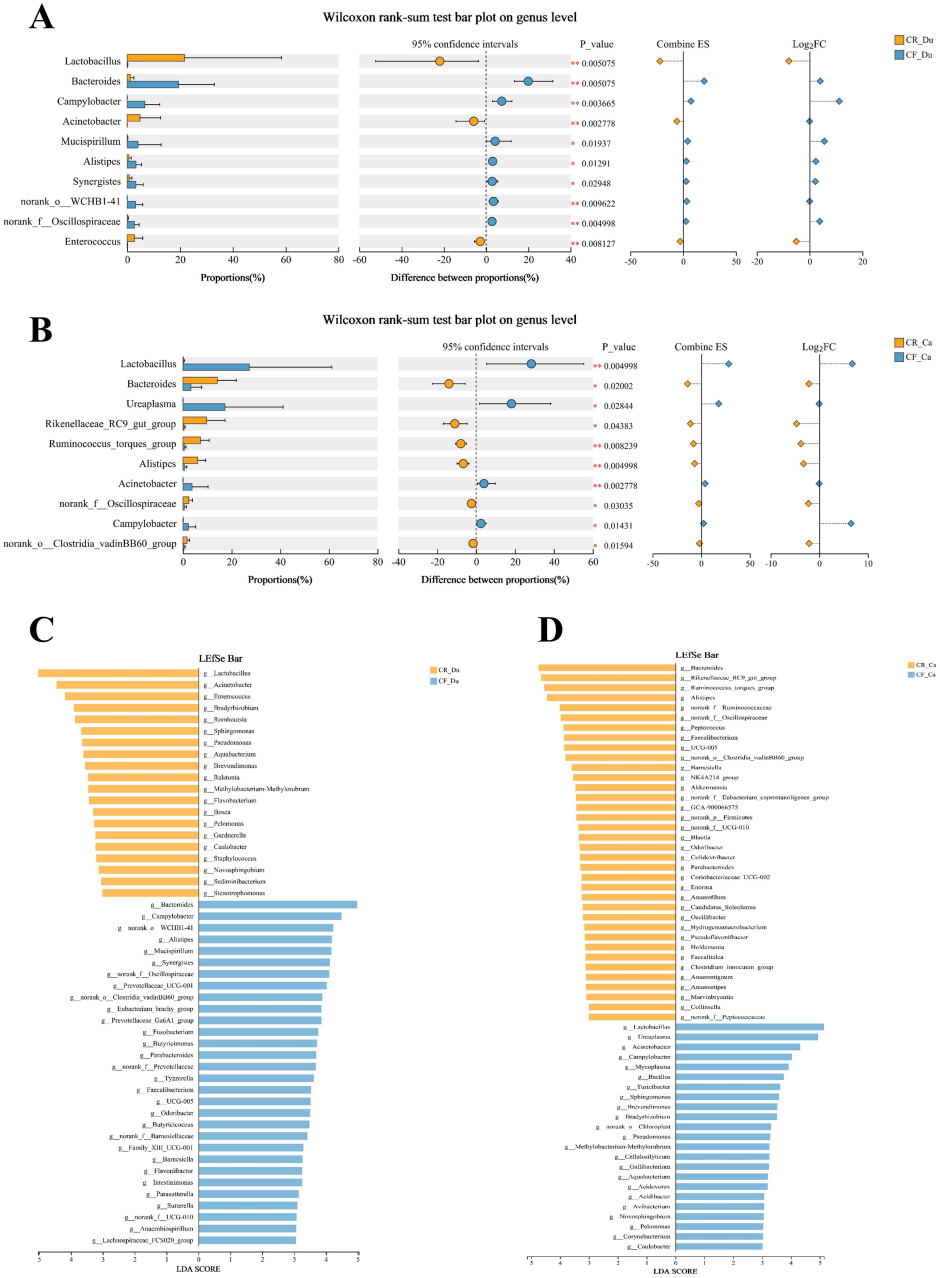
Differences in the composition of the gut microbiota of Lueyang black-bone chickens under different rearing systems. **(A,B)** Comparison of gut microbiota in the duodenum and cecum of Lueyang black-bone chickens under different rearing systems at the genus level; **(C,D)** LDA histogram of the duodenum and cecum microbiota under different rearing systems at the genus level (LDA value > 3.0, *p* < 0.05).

Intergroup differences in gut microbiota abundance between groups were assessed using difference testing and Lefse (Linear Discriminant Analysis Effect Size) analysis at the genus level (LDA value > 3.0, *p* < 0.05). In the duodenum, *Lactobacillus*, *Acinetobacter*, *Enterococcus*, *Romboutsia*, *Sphingomonas*, and *Pseudomonas* were significantly more abundant in the caged rearing group than in the cage-free rearing group, with statistical significance (Figures 4A,C; *p* < 0.05). *Lactobacillus*, *Acinetobacter*, and *Enterococcus* exhibited extremely significant differences (*p* < 0.01). In the cage-free rearing group, *Bacteroides*, *Campylobacter*, *Alistipes*, *Eubacterium*, and *Synergistes* were significantly more abundant than in the caged rearing group, with statistical significance (*p* < 0.05). *Bacteroides* and *Campylobacter* showed highly significant differences (*p* < 0.01).

In the cecum, *Bacteroides*, *Rikenellaceae_RC9_gut_group*, *Ruminococcus_torques_group*, *Alistipes*, *Peptococcus*, and *Faecalibacterium* were enriched in the caged rearing group, with statistical significance (Figures 4B,D; *p* < 0.05). *Ruminococcus_torques_group* and *Alistipes* exhibited extremely significant differences (*p* < 0.01). *Lactobacillus*, *Ureaplasma*, *Acinetobacter*, *Campylobacter*, *Bacillus*, *Mycoplasma*, and *Sphingomonas* were statistically significant (*p* < 0.05) in the cage-free rearing group, with *Lactobacillus* and *Acinetobacter* showing extremely substantial differences (*p* < 0.01).

### 3.5 Effects of different rearing systems on gut metabolism in Lueyang black-bone chickens

**Figure 5:**
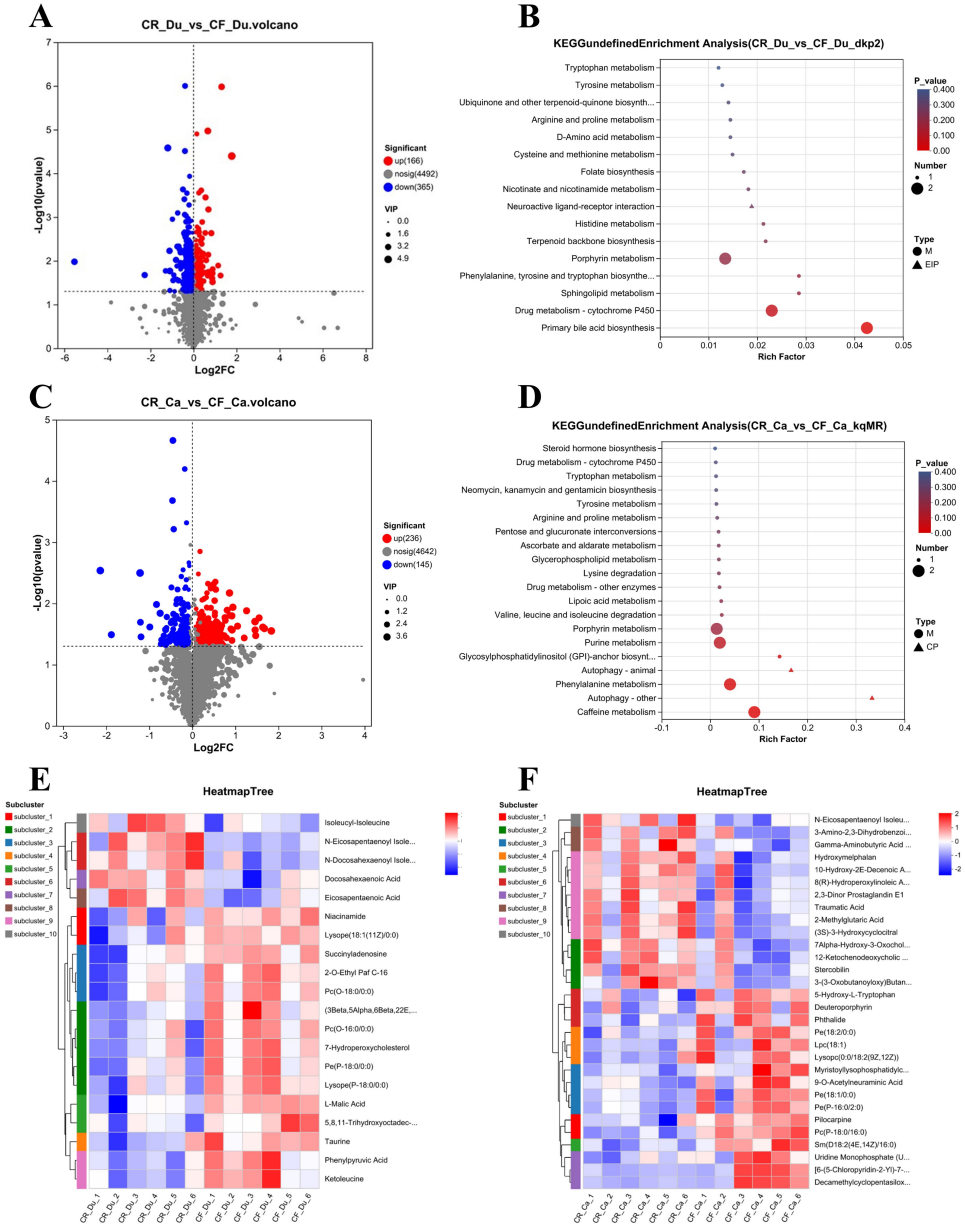
Metabolic profiles of the duodenum and cecum in Lueyang black-bone chickens under different rearing systems. **(A)** Volcano plot of metabolites in the duodenum under different rearing systems; **(B)** KEGG pathway enrichment analysis of differentially expressed metabolites in the duodenum under different rearing systems (top 20, *p* < 0.05); **(C)** Volcano plot of metabolites in the cecum under different rearing systems; **(D)** KEGG pathway enrichment analysis of differentially expressed metabolites in the cecum under different rearing systems (top 20, *p* < 0.05); **(E,F)** Cluster heatmap of differentially expressed metabolites in the duodenum and cecum under different rearing systems (top 20, *p* < 0.05).

OPLS-DA analysis was used to analyze and screen metabolite differences between the two groups. The default filter for differential metabolites uses *p* < 0.05, OPLS-DA VIP > 1, and up/down regulation differential multiples of 1. The results showed that 362 metabolites were significantly upregulated in the duodenum, 4,497 metabolites were significantly downregulated, and 170 metabolites exhibited no significant changes (Figure 5A). In the cecum, 145 metabolites were upregulated considerably, 4,568 were significantly downregulated, and 236 metabolites showed no significant changes (Figure 5C). These important metabolite changes may be associated with differences in metabolic activity in the cecum under different rearing systems.

KEGG pathway enrichment (top 20) analysis in the duodenum found that significant metabolites were primarily involved in alanine, aspartic acid, and glutamic acid metabolism, nicotinic acid and nicotinamide metabolism, and arginine biosynthesis pathways. Enriching these pathways suggests that different rearing systems also significantly impact metabolic activity in the duodenum, particularly amino acid and nucleotide metabolism (Figure 5B). In the duodenum, metabolites upregulated in the caged rearing group primarily included amino acid derivatives such as isoleucyl-isoleucine, N-eicosapentaenoyl isoleucine, and N-docosahexaenoyl isoleucine, as well as unsaturated fatty acids like docosahexaenoic acid (DHA) and eicosapentaenoic acid (EPA). In the cage-free rearing group, metabolites upregulated primarily included phospholipids and their derivatives such as 2-O-Ethyl Paf C-16, Pc (O-18:0/0:0), Pc (O-16:0/0:0), Pe (P-18:0/0:0), hemolytic phospholipids like Lysope (18:1(11Z)/0:0), Lysope (P-18:0/0:0), gut microbiota metabolites such as Ketoleucine and Phenylpyruvic acid, nucleotide metabolites like Succinyladenosine, cholesterol metabolites such as 7-Hydroperoxycholesterol, and anti-inflammatory agents such as Taurine and 5,8,11-trihydroxyoctadec-9-enoyl acid (Figure 5E).

KEGG pathway enrichment analysis (top 20) of differential metabolites in the cecum under different rearing systems revealed that significant metabolites in the cecum were primarily involved in pathways such as primary bile acid biosynthesis, histidine metabolism, lipid metabolism, and amino acid metabolism. The enrichment of these pathways suggests that different rearing systems significantly impact the metabolic activity of the cecum, particularly on bile acid and amino acid metabolism (Figure 5D). Cluster analysis of differential metabolites showed that in the cecum, metabolites upregulated in the caged rearing group included unsaturated fatty acids such as N-Eicosapentaenoyl Isoleucine, Traumatic Acid, and bile acids and their derivatives, such as Stercobilin and 7Alpha-Hydroxy-3-Oxochol-4-En-24-Oic Acid. In the cage-free rearing group, metabolites upregulated included phospholipids and their derivatives, such as Pe(18:2/0:0), Pe(18:1/0:0), Pc(P-18:0/16:0), Sm(D18:2(4E,14Z)/16:0), anti-inflammatory agents like phthalide, and hemolytic phospholipids such as Lpc(18:1), Lysopc(0:0/18:2(9Z,12Z)), and Myristoyllysophosphatidylcholine (Figure 5F).

### 3.6 Correlation analysis between gut microbiota and gut metabolism in Lueyang black-bone chickens under different rearing systems

**Figure 6:**
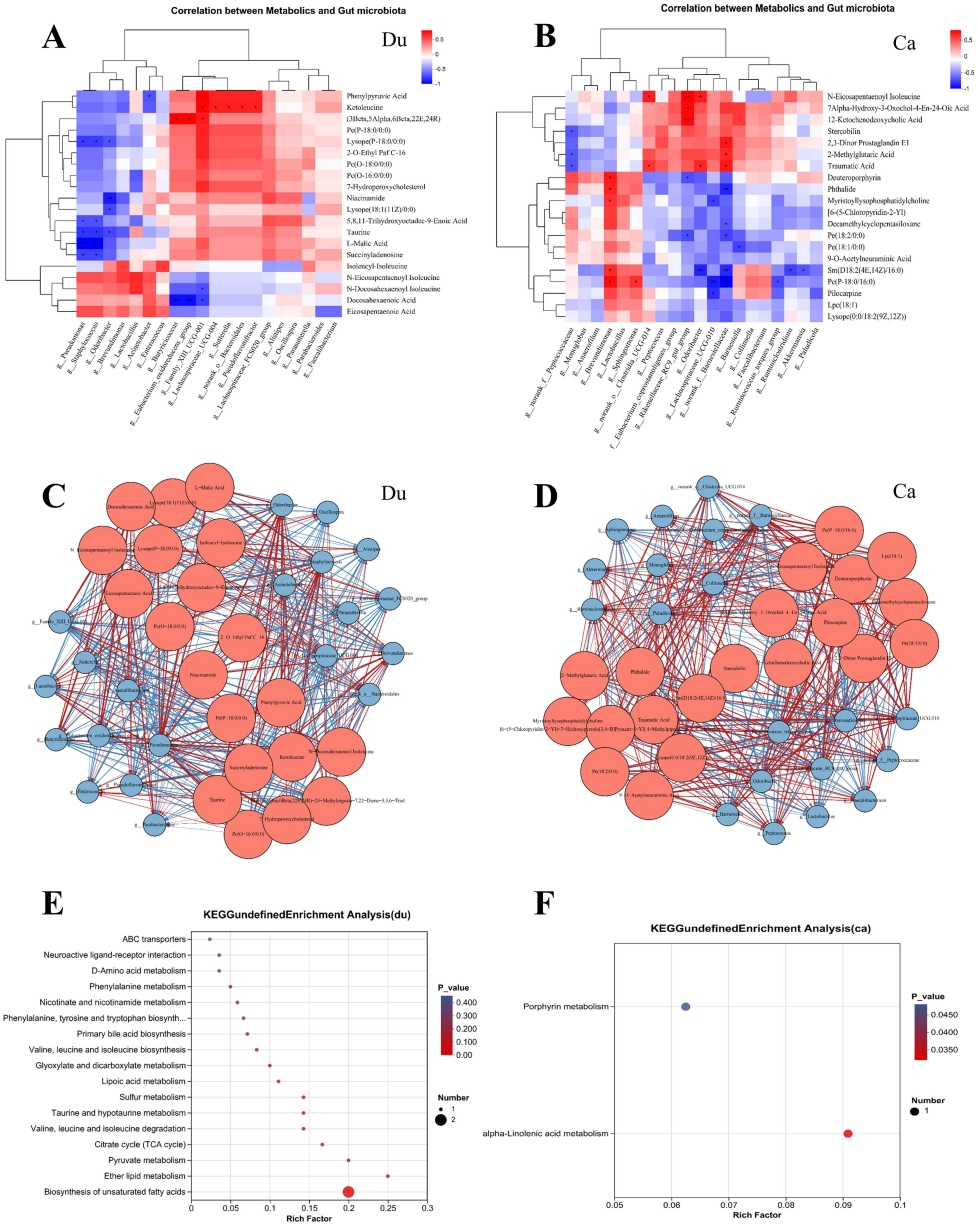
Correlation analysis between gut microbiota and gut metabolism in Lueyang black-bone chickens under different rearing systems. **(A,B)** Heatmap correlation between differential microbes and metabolites in the duodenum and cecum under different rearing systems. The depth of color indicates the magnitude of the correlation coefficient, with red and blue representing positive and negative correlations, respectively. Black stars indicate significant correlations: *p < 0.05, **p < 0.01, ***p < 0.001; **(C,D)** Correlation network diagram of differential gut microbiota and metabolites in Lueyang black-bone chickens under different rearing systems. Red nodes represent metabolites, blue nodes represent microorganisms, and node size indicates the number of nodes with significant correlations. Red lines represent positive correlations, while blue lines represent negative correlations. **(E,F)** KEGG pathway enrichment analysis of the top 20 metabolites strongly correlated with differential microorganisms.

Spearman correlation analysis (Figures 6A,B) and network analysis (Figures 6C,D) were performed to explore the relationship between the gut microbiota, metabolites, and intestinal barrier stability in Lueyang black-bone chickens under different rearing systems, based on the cecal and duodenal microbiota and metabolomics data mentioned above. This study focused on the top 20 differential microbiota and the top 20 metabolites for correlation analysis.

In the duodenum, the correlation heatmap shows that *Butyricicoccus* and *Eubacterium_oxidoreducens_group* were significantly positively correlated with (3Beta,5Alpha,6Beta,22E,24R)-23-Methylergosta-7,22-Diene-3,5,6-Triol; *Family_XIII_UCG-001* was significantly positively correlated with Phenylpyruvic Acid, Ketoleucine, and (3Beta,5Alpha,6Beta,22E,24R)-23-Methylergosta-7,22-Diene-3,5,6-Triol, and significantly negatively correlated with N-Docosahexaenoyl Isoleucine and Docosahexaenoic Acid; *Lachnospiraceae_UCG-004*, *Sutterella*, *Bacteroidales*, and *Pseudoflavonifractor* were significantly positively correlated with Ketoleucine. Further KEGG pathway enrichment analysis of several highly correlated differential metabolites revealed that changes in rearing systems significantly affected the biosynthesis of unsaturated fatty acids (*p* < 0.01) and Ether lipid metabolism (*p* < 0.05) in the duodenum (Figure 6E).

The correlation heatmap in the cecum shows *Brevundimonas* is significantly positively correlated with Deuteroporphyrin, Phthalide, Myristoyllysophosphatidylcholine, Sm(D18:2(4E,14Z)/16:0), Pc(P-18:0/16:0); *Clostridia_UCG-014* is significantly positively correlated with N-Eicosapentaenoyl Isoleucine, Traumatic Acid; *Rikenellaceae_RC9_gut_group* is significantly positively correlated with N-Eicosapentaenoyl Isoleucine, Alpha-Hydroxy-3-Oxochol-4-En-24-Oic Acid, nd12-Ketochenodeoxycholic Acid *Odoribacter* showed a significant positive correlation with N-Eicosapentaenoyl Isoleucine and Traumatic Acid; *Lachnospiraceae_UCG-010* showed a significant negative correlation with Myristoyllysophosphatidylcholine, Pc(P-18:0/16:0), and Pilocarpine. Further KEGG pathway enrichment analysis of several differentially expressed metabolites with strong correlations revealed that changes in rearing systems significantly affected two metabolic pathways in the cecum (p < 0.05): Porphyrin metabolism and *α*-linolenic acid metabolism (Figure 6F).

## 4 Discussion

### 4.1 Effects of different rearing systems on the gut morphology of Lueyang black-bone chickens

The integrity of gut barrier function is a fundamental physiological mechanism for maintaining immune homeostasis, nutrient absorption, and microbial balance. Numerous studies have demonstrated that intestinal wall thickness, goblet cell count, and the villus-to-crypt depth ratio are critical morphological indicators for assessing gut barrier integrity. Intestinal wall thickness is influenced by MUC2, secreted by goblet cells in the mucosal layer, and tight junction proteins, ZO-1 and Claudin-1. A thickened mucosal layer enhances the physical barrier function of the intestine, thereby maintaining intestinal barrier homeostasis (Sertaridou et al., 2015; Cukrowska et al., 2017). Dynamic changes in the villus height (Vh) to crypt depth (Cd) ratio (V/C ratio) directly reflect the gut’s absorption capacity, regenerative potential, and barrier integrity. Studies have demonstrated that the V/C ratio generally correlates positively with the expression of tight junction proteins, ZO-1 and Claudin-1. For instance, in a pig model, oxidative stress resulted in a significant decrease in the V/C ratio of the small intestine, along with reduced mRNA and protein expression levels of ZO-1 and Claudin-1(Hu et al., 2021). In this study, tissue section analysis revealed that intestinal wall thickness, goblet cell count, and V/C ratio in the cecum and duodenum of the caged rearing group were significantly higher than those in the cage-free rearing group, indicating enhanced mucosal defense capacity and suggesting a more stable intestinal barrier function in the caged rearing group. In the cage-free rearing group, animals were exposed to complex microbial communities in soil and water sources, increasing their risk of pathogen exposure and subjecting gut cells to greater damage (Hubert et al., 2019). For instance, the pathogenic bacterium *Campylobacter* secretes the toxin LOS, which activates the host caspase signaling pathway, induces apoptosis of gut epithelial cells, and causes redistribution of tight junction proteins, Claudin-5 and Occludin, forming “leaky foci”(Harrer et al., 2019). Therefore, caged rearing promotes gut microbiota homeostasis by reducing external microbial interference.

### 4.2 Effects of different rearing systems on the microbial diversity of gut microbiota in Lueyang black-bone chickens

It has been shown that rearing systems influence the diversity of microorganisms in the gut of chickens. This study found that the abundance of probiotics, such as *Lactobacillus* and *Enterococcus*, was significantly upregulated in the duodenum of the caged rearing group. *Lactobacillus* enhanced the transmembrane resistance (TER) of Caco-2 cell monolayers and inhibited barrier disruption induced by the pathogen ETEC (Karimi et al., 2018). In a necrotizing enterocolitis (NEC) model, *Lactobacillus* reduced gut permeability and stabilized the expression of ZO-1 protein (Blackwood et al., 2017). *Enterococcus* exerts a dual effect on the intestinal barrier. *Enterococcus faecalis* secretes mucinase to degrade MUC2, weakening the gut’s physical barrier and increasing the risk of pathogen colonization (Ferchichi et al., 2021). *Enterococcus faecium NCIMB 10415* also activates the calmodulin signaling pathway to enhance the transmembrane resistance (TER) of the intestinal epithelial cell monolayer, which helps maintain the structural integrity of tight junctions between intestinal epithelial cells (Klingspor et al., 2015). It is therefore speculated that the increase in *Lactobacillus* and *Enterococcus* in the duodenum of caged rearing animals contributes to maintaining intestinal barrier homeostasis. The relative abundance of *Bacteroides* and *Campylobacter* was significantly increased in the cage-free rearing group. *Campylobacter* is a Gram-negative aerobic bacterium that directly destroys the integrity of the gut barrier by secreting HtrA protease to degrade the tight junction protein ZO-1(Heimesaat et al., 2023). In human studies, *Campylobacter jejuni* infection of HT-29 human colon cancer cells significantly upregulates pro-apoptotic proteins Bax and Bid, downregulates anti-apoptotic protein Bcl-2, and reduces the expression of the goblet cell marker MUC2 (Butkevych et al., 2020). Therefore, the reduction in goblet cell numbers in the duodenum and cecum of cage-free rearing animals may be associated with *Campylobacter*, which impairs the maintenance of the gut barrier.

This study found significant enrichment of *Bacteroides*, *Rikenellaceae_RC9_gut_group*, and *Alistipes* in the cecum of the caged rearing group. The study showed that *Bacteroides* degrades dietary fiber to produce butyrate, which activates peroxisome proliferator-activated receptor gamma (PPAR-*γ*), induces MUC2 mucin synthesis, increases mucus layer thickness, and reduces pathogen penetration (El-Sayed et al., 2024; Zhou et al., 2024). *Rikenellaceae_RC9_gut_group* degrades fiber into oligosaccharides, ultimately producing SCFAs that reduce pro-inflammatory factors such as IL-1β and IL-6, thereby reducing inflammation-induced damage to tight junction proteins (Parada Venegas et al., 2019; Gonçalves-Nobre et al., 2023). A study of Tibetan sheep showed that *Ruminococcus* enhances energy metabolism by promoting SCFA absorption, supporting the regeneration of epithelial cells in the gut (Chen et al., 2024). *Alistipes* metabolizes tryptophan into products, such as indole-3-acetic acid, activates the aromatic hydrocarbon receptor AhR, induces IL-22 secretion, promotes mucus production and epithelial repair, and supports gut barrier formation (Zelante et al., 2013). Therefore, the enrichment of *Bacteroides*, *Rikenellaceae_RC9_gut_group*, and *Alistipes* contributes to maintaining the cecal barrier in the caged rearing group. However, *Campylobacter* and *Acinetobacter* were significantly enriched in the cecum of the cage-free rearing group. Studies have shown that *Campylobacter* colonization is associated with increased IL-8 secretion in the intestinal epithelium and neutrophil infiltration, promoting inflammation and inhibiting the repair of intestinal epithelial tissue (Esson et al., 2016). *Acinetobacter* induces chronic inflammation, leading to abnormal proliferation of crypt basal stem cells, formation of adenomatous structures, and weakening of the intestinal barrier (Korinek et al., 1997; Klingler et al., 2022). Therefore, it can be inferred that the enrichment of *Campylobacter* and *Acinetobacter* in the cecum reflects an imbalance and instability in the microbial community structure of the gut in cage-free reared animals, indicating a diseased state.

### 4.3 Correlation between core gut microbiota and metabolites in Lueyang black-bone chickens under different rearing systems

Host health regulation by the microbiota is primarily mediated through the release of common intermediate metabolites. In the duodenum, omega-3 unsaturated fatty acids, including docosahexaenoic acid (DHA) and eicosapentaenoic acid (EPA), were significantly enriched in the caged rearing group. DHA and EPA significantly enhanced the Muc2 secretion capacity of LS174T goblet cells by alleviating palmitic acid (PAL)-induced endoplasmic reticulum stress (ERS). DHA also inhibits the RIPK1/RIPK3/MLKL necrotic apoptosis signaling pathway, reduces TNF-*α*-induced tight junction (TJ) damage in porcine intestinal epithelial cells (IPEC-1), and upregulates the expression of Claudin-1 and Occludin, thereby maintaining gut barrier homeostasis (Escoula et al., 2019; Xiao et al., 2020). In the cecum, metabolites enriched in the caged rearing group included the unsaturated fatty acid N-Eicosapentaenoyl Isoleucine (NEI), a conjugated derivative of omega-3 polyunsaturated fatty acids (EPA). NEI can be metabolized into PGE3 and other dissipation mediators, selectively inhibiting COX-2 activity and reducing the production of pro-inflammatory PGE2. In inflammatory bowel disease (IBD), elevated PGE2 levels exacerbate gut barrier damage. At the same time, PGE3 inhibits NF-κB and MAPK signaling pathways by antagonizing the PGE2 receptor EP4, thereby reducing the expression of pro-inflammatory factors such as TNF-α and IL-6 (Rodríguez-Lagunas et al., 2013). LysoPC, enriched in the cage-free rearing group, is a bioactive lipid produced by phospholipase hydrolysis, containing a monounsaturated chain and a polar head. In LPS-induced gut inflammation models, lysophospholipids (such as LysoPC) activate the TLR4/NF-κB pathway, upregulate TNF-α and IL-1*β* expression, and exacerbate gut barrier damage (Carneiro et al., 2013). In summary, this study found that the unsaturated fatty acid levels were significantly higher in the caged rearing group compared to the cage-free rearing group. Additionally, morphological findings from tissue sections revealed that the caged rearing group exhibited substantially higher intestinal barrier-related indicators than the cage-free rearing group. Therefore, it can be inferred that the significant enrichment of unsaturated fatty acids under caged rearing conditions plays a key role in maintaining the integrity of the intestinal barrier in the caged rearing group.

Further correlation analysis revealed a significant positive correlation between *Rikenellaceae_RC9_gut_group* in the cecum and the metabolites 7Alpha-Hydroxy-3-Oxochol-4-En-24-Oic Acid and 12-Ketochenodeoxycholic Acid. Both metabolites are intermediates in secondary bile acid metabolism. Bile acids are end products of cholesterol metabolism in the liver and are classified into primary and secondary forms. Primary bile acids are synthesized directly by liver cells, while secondary bile acids are produced by the gut microbiota (Wahlström et al., 2016). Studies have shown that *Rikenellaceae_RC9_gut_group* can convert primary bile acids into secondary bile acids via 7α-dehydroxylase, promoting the secretion of MUC2 mucin by goblet cells, enhancing the mucus barrier, and regulating bile acid circulation (Kim et al., 2022). Additionally, *Clostridia_UCG-014* exhibits a significant positive correlation with the metabolite Traumatic Acid. *Clostridia_UCG-014* is closely associated with the tryptophan metabolic pathway, producing indole derivatives (IAA) through tryptophan breakdown, which activates the aryl hydrocarbon receptor (AhR) and regulates IL-22 secretion (Kemter et al., 2023). Traumatic Acid is an unsaturated fatty acid derivative that neutralizes free radicals and enhances cellular oxidative stress defense. In human skin fibroblasts, Traumatic Acid activates the TGF-β/Smad pathway, upregulates the expression of type I and III collagen genes, significantly increases collagen secretion, and accelerates wound healing (Jabłońska-Trypuć et al., 2016). In summary, caged rearing may maintain gut barrier homeostasis by upregulating gut microbes such as *Lactobacillus*, *Enterococcus*, *Rikenellaceae_RC9_gut_group*, *Alistipes*, and others, as well as regulating metabolites such as unsaturated fatty acids and bile acid metabolites.

## 5 Conclusion

The differential regulatory mechanisms of the gut barrier in Lueyang black-bone chickens reared in caged and cage-free conditions were investigated. High-throughput sequencing of gut 16S rRNA and LC–MS-based non-targeted metabolomics analysis were integrated to study changes in the gut microbiota and metabolome of the caged and cage-free rearing groups, along with the interactions between microorganisms and metabolites. This study provides a theoretical foundation for optimizing poultry rearing strategies based on the interactions between microorganisms and metabolites.

## Data Availability

The original contributions presented in the study are publicly available. This data can be found here: https://www.ncbi.nlm.nih.gov/, accession number PRJNA1308479.

## Footnotes

^1^ http://huttenhower.sph.harvard.edu/LEfSe

^2^ http://www.hmdb.ca/

^3^ https://metlin.scripps.edu/

^4^ https://www.kegg.jp/kegg/pathway.html
